# Nanoscale anisotropic plastic deformation in single crystal GaN

**DOI:** 10.1186/1556-276X-7-150

**Published:** 2012-02-22

**Authors:** Jun Huang, Ke Xu, Ying Min Fan, Mu Tong Niu, Xiong Hui Zeng, Jian Feng Wang, Hui Yang

**Affiliations:** 1 Suzhou Institute of Nano-tech and Nano-bionics, Chinese Academy of Sciences, Ruoshui Road 398, Suzhou, 215123, People's Republic of China

**Keywords:** GaN, anisotropic, hydride vapor phase epitaxy, nanoindentation, cathodoluminescence, transmission electron microscopy.

## Abstract

Elasto-plastic mechanical deformation behaviors of c-plane (0001) and nonpolar GaN single crystals are studied using nanoindentation, cathodoluminescence, and transmission electron microscopy. Nanoindentation tests show that c-plane GaN is less susceptible to plastic deformation and has higher hardness and Young's modulus than the nonpolar GaN. Cathodoluminescence and transmission electron microscopy characterizations of indent-induced plastic deformation reveal that there are two primary slip systems for the c-plane GaN, while there is only one most favorable slip system for the nonplane GaN. We suggest that the anisotropic elasto-plastic mechanical properties of GaN are relative to its anisotropic plastic deformation behavior.

**PACS: **62.20.fq; 81.05.Ea; 61.72.Lk.

## Introduction

GaN-related III-nitride materials are attracting increasing attention for a wide range of device applications, such as light-emitting diodes [1], blue laser devices [2], and high electron mobility transistor [3]. Besides, GaN also exhibits attractive mechanical properties for potential device manufacture such as making microelectromechanical systems and making surface acoustic wave device [4,5]. Therefore, there is an increasingly growing interest in the elasto-plastic mechanical behaviors of GaN material [6-16].

The previous investigations in the elasto-plastic mechanical behaviors of GaN were mainly carried out on a c-plane GaN. The only few works performed on nonpolar ((11-20) and (10-10) planes) GaN epitaxial layers, however, were largely affected by the substrate or the growth induced stacking faults [6-16]. Moreover, despite it is well known that GaN crystallizes in the wurtzite structure is characterized by a high anisotropy, a study on anisotropic elasto-plastic mechanical behaviors of GaN is still lacking.

This work attempts to present nanoindentation experiments performed on the three principal surfaces, (0001), (11-20), and (10-10), of GaN single crystals. Furthermore, the underlying activated slip systems which govern the plastic deformation of GaN are identified by cathodoluminescence (CL) and transmission electron microscopy. Finally, a comparison between the c-plane and nonpolar GaN in mechanical properties and plastic deformation is presented.

## Experimental details

The samples under investigations were grown by hydride vapor phase epitaxy in homemade reactor. The c-plane GaN samples were obtained from a 1.5-mm thick free standing GaN wafer with dislocation density lower than 10^6 ^cm^-2^. Also, the a-plane and m-plane samples were obtained from a 1.5-mm thick free standing GaN wafer by cleaving along the (11-20) and (10-10) planes, respectively. The crystal orientations were identified by X-ray diffraction. The mechanical properties of GaN crystals were conducted with a nanoindentation system (Nano indenter G200, Agilent Technologies Inc., Santa Clara, CA, USA). The analytical method developed by Oliver and Pharr [17] was adopted to determine the hardness (H) and Young's modulus (E) of the GaN crystals from indentations with a maximum penetration depth of 500 nm. Scanning electron microscopy (Quanta 400 FEG, FEI Company, Hillsboro, OR, USA) and its attached cathodoluminescence (MonoCL3+) (SEM-CL) were combined to characterize the indentation-induced plastic deformation at room temperature. The cross-sectional transmission electron microscopy (XTEM) samples were prepared from the indents by means of a dual-beam focused ion beam (Nova 220) station with Ga ions. A FEI Tecnai G2 F20 S-Twin TEM operated at an accelerating voltage of 200 kV was used to study the microstructures of obtained XTEM samples.

## Results and discussion

**Figure 1:**
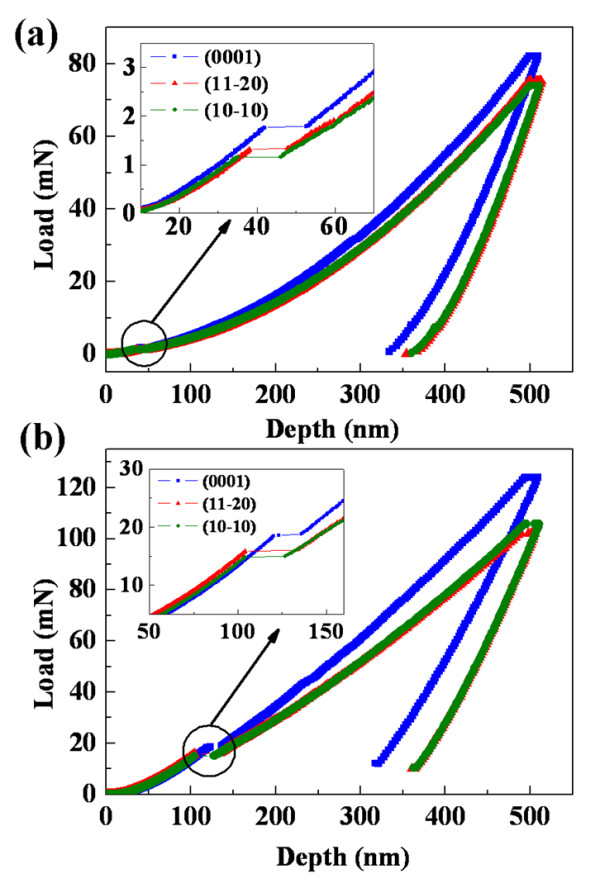
**Typical continuous load-depth data**. Data obtained from c-plane (0001), a-plane (11-20), and m-plane (10-10) GaN crystals performed with a Berkovich tip (**a**) and a conical tip (**b**). The insets are the magnified views of the pop-in events.

Figure 1a, b depicts the averaged nanoindentation load-depth data of GaN single crystals using a Berkovich diamond tip (three-sided pyramid with a nominal tip radius of 50 nm) and a conical diamond tip (90°, 5-μm radius), respectively. Nanoindentation tests were performed on three mutually orthogonal surfaces, where one surface is the hexagonal crystal c-plane and the other two surfaces ((10-10) and (11-20) planes) are normal to the c-plane. Obviously, each curve exhibits a single discontinuity (also referred as 'pop-in') that marks incipient plasticity during loading. Evidently, before the occurrence of pop-in event (the elastic regime), the load-depth curves of three orthogonal surfaces are almost superimposed on each other. However, after the occurrence of pop-in event (the elasto-plastic regime), the slopes of the load-depth curves of nonpolar GaN are smaller than that of the c-plane GaN, indicating an anisotropic plastic deformation behavior of GaN.

Applying the Oliver and Pharr method [17] to the indentations with the maximum penetration depth of 500 nm (Figure 1), the Young's modulus and the hardness of GaN were determined as shown in Table 1. Poisson's ratio of 0.183 was adopted in the present study according to Moram et al. [18]. Each sample was measured more than 20 indentations to make an average value. Also, the mean square error of each data is given in Table 1. Obviously, the E and H of c-plane GaN are larger than that of the nonpolar GaN, and these anisotropic mechanical properties of GaN are independent of the tip geometry.

**Table 1:** Indenter tip, contact plane, Young's modulus (E), and hardness (H) of GaN single crystal

Indenter tip	Plane	E (GPa)	H (GPa)
Berkovich tip	(0001)	333.61 ± 2.70	19.04 ± 0.23
	(11-20)	330.56 ± 2.72	15.31 ± 0.20
	(10-10)	329.74 ± 3.26	15.24 ± 0.21
Conical tip	(0001)	317.34 ± 4.12	20.16 ± 0.48
	(11-20)	308.26 ± 3.82	15.87 ± 0.41
	(10-10)	306.96 ± 3.98	16.23 ± 0.44

Values were obtained from nanoindentation tests on GaN single crystal. The data from the indentation tests represent average data from at least 20 valid initial values. Also, the mean square error of each average data is given in the table. Values of E and H were calculated by Oliver and Pharr method [17] using load-depth curve of Figure 1.

In order to fully understand the deformation mechanism of c-plane GaN and nonpolar GaN under nanoindentation, the damages induced by conical tip were investigated by CL and XTEM (the damages induced by Berkovich tip is similar to but have less symmetry than those induced by conical tip).

**Figure 2:**
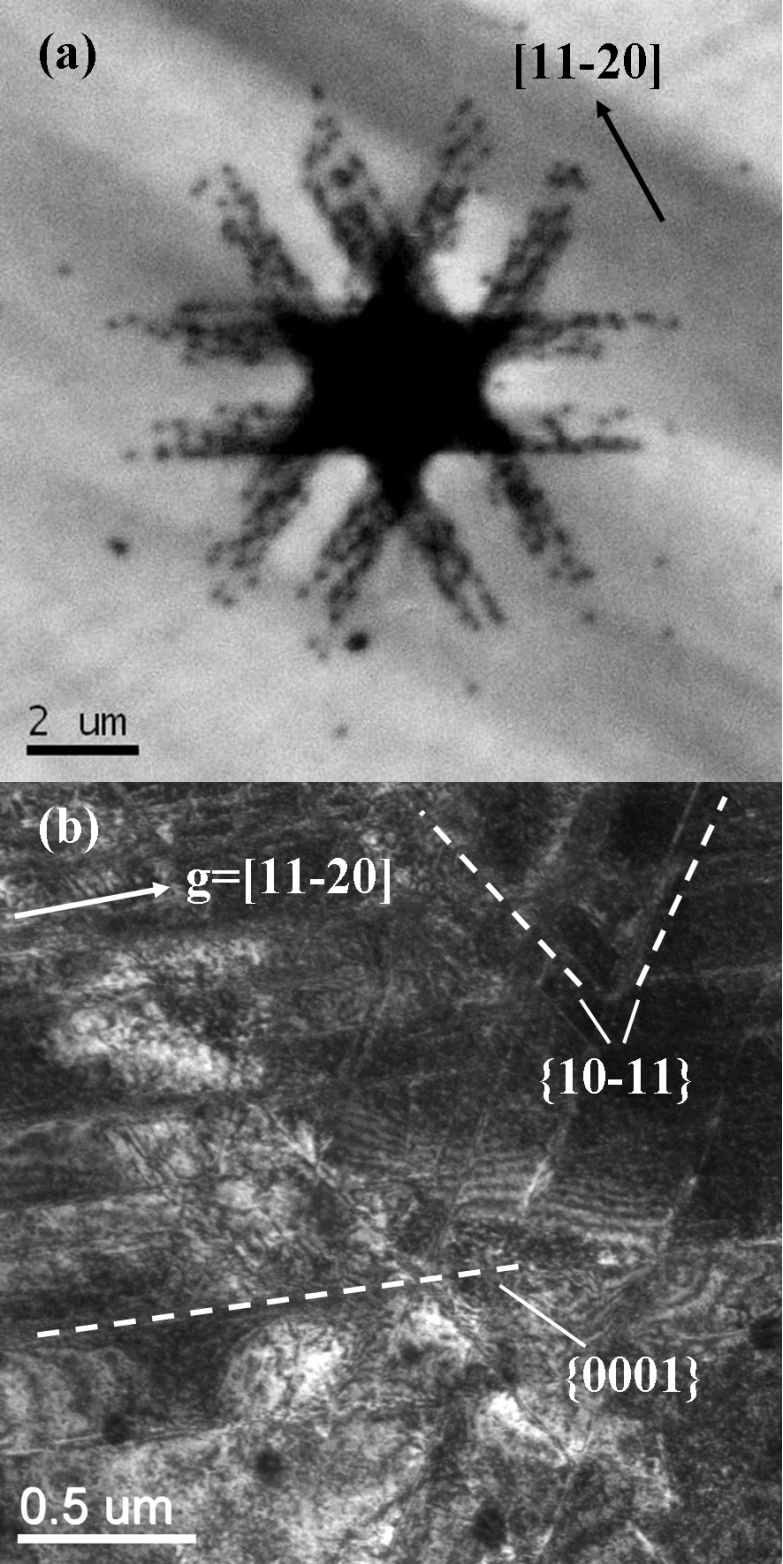
**Room-temperature panchromatic CL images and dark-field XTEM image in c-plane GaN**. (**a**) Room-temperature panchromatic CL images of a conical indent in c-plane GaN. CL imaging condition, electron beam energy = 5 keV. (**b**) Dark-field XTEM image of the region beneath a conical indent in c-plane GaN.

Figure 2a shows CL images of the c-plane indented regions at a maximun penetration depth of 500 nm. The contact-induced extended defects can be observed to extend radially out from the center of the indentation along < 11-20 > crystal orientation, a characteristic of the hexagonal crystal system. Nevertheless, CL imaging of GaN indented below the critical pop-in load revealed no detectable reduction in the intensity of CL emission, indicating that the pop-in event involves the nucleation of dislocation as the deformation mode.

To further elucidate the slip band of nanoindentation-induced deformation, an indent with maximum depth of 500 nm in c-plane GaN is investigated by XTEM. Figure 2b displayed a dark-field XTEM picture in the vicinity of the indented region. The picture displays a microstructure of the slip bands, characterized by features of aligned bright lines in the photograph. It can be seen clearly that the slip bands aligned parallel to the (0001) basal plane intersect the slip bands propagating along the (10-11) pyramidal plane. The two sets of slip bands suggest that during the indentation, the rapidly increasing dislocations can glide collectively along those planes. Moreover, selected area diffraction patterns of the highly damaged regions directly beneath the indenter showed no evidence of structural transformations to other phases of GaN. Therefore, the dislocations gliding along both the (0001) basal planes and the (10-11) pyramidal planes should be the primary mechanism of plastic deformation on the c-plane GaN during loading. The result is consistent with the previous reports [6,8,10-12].

**Figure 3:**
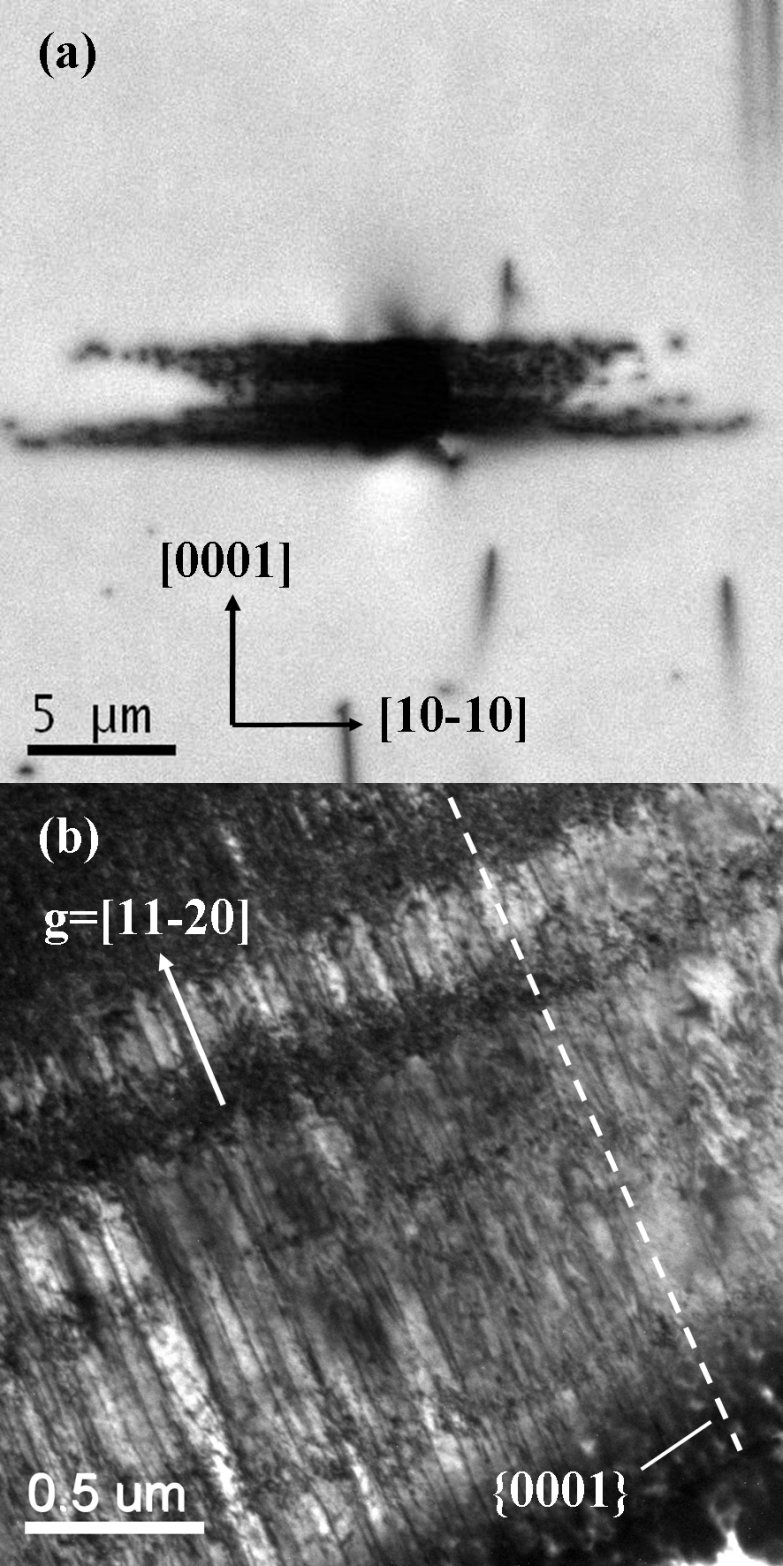
**Room-temperature panchromatic CL images and dark-field XTEM image in a-plane GaN**. (**a**) Room-temperature panchromatic CL images of conical indent in a-plane GaN. CL imaging condition, electron beam energy = 5 keV. (**b**) Dark-field XTEM image of the region beneath a conical indent in a-plane GaN.

Furthermore, CL and XTEM images of the conical indentations in a-plane GaN with maximum penetration depth of 500 nm are illustrated in Figure 3. The extent of the aligned propagation of contact-induced defects can be seen clearly in CL image of Figure 3a. In contrast to the plastic deformation pattern with a sixfold structure symmetry on c-plane GaN, the contact-induced defects on a-plane GaN are extended exclusively out from the center of the indentations along the (10-10) direction. The discrepancy indicates that there should be a different deformation mechanism for a-plane GaN. Figure 3b displays dark-field XTEM image of the indentation-induced damage in a-plane GaN. Apparently, only slip bands propagating along the (0001) basal plane can be found, and the (10-11) pyramidal slip planes have not been found in this case. In addition, indentation in m-plane GaN shows a similar result to a-plane GaN, namely the principal slip planes of m-plane GaN during indentation are also (0001) basal planes. Therefore, CL and XTEM results of indentation-induced damage reveal an anisotropic plastic deformation behavior of GaN.

## Conclusions

To summarize, the anisotropic mechanical properties and deformation behavior based on high-quality GaN bulk material were presented in this paper. Nanoindentation tests reveal that nonpolar GaN has lower values than the c-plane GaN in both Young's modulus and hardness. Also, CL and XTEM measurements show that there are two primary dislocation slip planes ((0001) and (10-11) planes) for c-plane GaN, while there is only one most favorable dislocation slip plane ((0001) plane) for nonplane GaN during plastic deformation. We suggest that the anisotropic elasto-plastic mechanical properties of GaN are relative to its anisotropic plastic deformation behavior.

## Competing interests

The authors declare that they have no competing interests.

## Authors' contributions

JH carried out all the experiments, participated in the sequence alignment, and drafted the manuscript. KX, JFW, and HY carried out the growth of GaN. YMF participated in the nanoindentation measurement. MTN participated in the TEM measurement and analysis. XHZ participated in the CL measurement. All authors read and approved the final manuscript.
